# Astrocyte and Oligodendrocyte Responses From the Subventricular Zone After Injury

**DOI:** 10.3389/fncel.2021.797553

**Published:** 2021-12-24

**Authors:** Jennifer David-Bercholz, Chay T. Kuo, Benjamin Deneen

**Affiliations:** ^1^Department of Cell Biology, Duke University School of Medicine, Durham, NC, United States; ^2^Department of Anesthesiology, Duke University School of Medicine, Durham, NC, United States; ^3^Center for Cell and Gene Therapy, Baylor College of Medicine, Houston, TX, United States; ^4^Department of Neuroscience, Baylor College of Medicine, Houston, TX, United States; ^5^Department of Neurosurgery, Baylor College of Medicine, Houston, TX, United States

**Keywords:** SVZ, neural stem cell, niche, astrocytes, oligodendrocytes, injury

## Abstract

Under normal conditions, neural stem cells (NSCs or B cells) in the adult subventricular zone (SVZ) give rise to amplifying neural progenitor cells (NPCs or C cells), which can produce neuroblasts (or A cells) that migrate to the olfactory bulb and differentiate into new neurons. However, following brain injury, these cells migrate toward the injury site where they differentiate into astrocytes and oligodendrocytes. In this review, we will focus on recent findings that chronicle how astrocytes and oligodendrocytes derived from SVZ-NSCs respond to different types of injury. We will also discuss molecular regulators of SVZ-NSC proliferation and their differentiation into astrocytes and oligodendrocytes. Overall, the goal of this review is to highlight how SVZ-NSCs respond to injury and to summarize the regulatory mechanisms that oversee their glial response. These molecular and cellular processes will provide critical insights needed to develop strategies to promote brain repair following injury using SVZ-NSCs.

## Introduction

In recent years, research has begun to unravel complex biological cascades that follows injury to the brain: astroglial activation and proliferation (Pekny et al., 2016, 2019), parenchymal inflammation and infiltration of immune cells (Liesz et al., 2015; Gill and Veltkamp, 2016; Sofroniew, 2020), glial scar formation (Burda et al., 2016), revascularization/re-establishment of blood-brain barrier (Li et al., 2021), remodeling of connections between surviving neurons (Jones, 2017) and replenishment of oligodendrocytes following demyelination injury (Nait-Oumesmar et al., 2007; Butti et al., 2019).

Research in this area has revealed new methods to improve and augment these responses after injury. One potentially exciting strategy is to enhance NSC proliferation in the postnatal/adult brain. During embryogenesis these NSC populations are relatively abundant, however, in the adult they become restricted to specialized regions/niches in the brain. Specifically, NSCs reside in the SVZ along the lateral walls of lateral brain ventricles (Ihrie and Álvarez-Buylla, 2011) and the subgranular zone (SGZ) of the hippocampal dentate gyrus (Bonaguidi et al., 2012). In the SVZ, NSCs (B cells) are located in the walls of the ventricular-SVZ and give rise to transit amplifying cells (NPC or C cells), which can produce neuroblasts (A cells) (Altman, 1962). Under physiological conditions, neuroblasts migrate a long-distance (3–8 mm in mice) from the SVZ to the olfactory bulb (Lois and Alvarez-Buylla, 1994; Lois et al., 1996), where they differentiate into granule neurons (Grelat et al., 2018; Li et al., 2018). Following injury such as ischemic stroke, neuroblast migration can be redirected from the rostral migratory stream (RMS)-olfactory bulb toward the site of injury (for review see Chang et al., 2016), suggesting that endogenous injury responses can mobilize NSCs from the SVZ niche.

In addition to neurons, NSCs also make astrocytes and oligodendrocytes. Several groups reported that following stroke, cells coming from the SVZ are predominantly glial instead of neuronal (Givogri et al., 2006; Li et al., 2010) and that these cells play a major role in glial scar formation following injury (Benner et al., 2013). The SVZ also plays a role in myelin repair and oligodendrocyte formation. After white matter injury, the number of oligodendrocytes derived from SVZ increases in the corpus callosum (CC) (Nait-Oumesmar et al., 1999; Picard-Riera et al., 2002; Menn et al., 2006).

**FIGURE 1:**
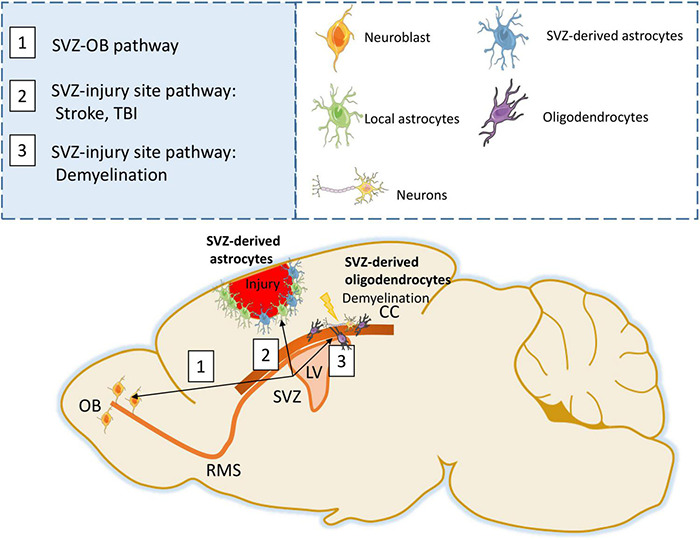
SVZ response following injury. Under physiological conditions, SVZ progenitors generate neuroblasts that migrate through the RMS to the Olfactory bulb (OB) (Path 1). Following stroke or TBI, SVZ progenitors are able to generate astrocytes that migrate to the injury site forming a glial scar (Path 2). Following demyelination, SVZ progenitors can differentiate into oligodendrocytes and participate in remyelination (Path 3).

Neurogenesis is a cardinal feature of SVZ-NSCs under homeostatic conditions, however after injury the differentiation programs of these SVZ-NSCs can be redirected toward the generation of glial cells (astrocytes and oligodendrocytes) (Figure 1). Therefore, in this review we will describe SVZ-glia contribution following different forms of brain injury, and discuss the factors regulating their function with the goal of identifying areas of potential therapeutic interest.

## Subventricular Zone-Derived Astrocytes Response to Injury

Under normal physiological conditions, the SVZ can generate mature astrocytes (Sohn et al., 2015) and following injury, the number of astrocytes produced by the SVZ drastically increases. Lineage tracing with the Nestin-CreER^T2^ promoter has been used to directly identify SVZ-derived progenitors migrating to the injury site, with the Nestin-CreER^T2^:R26R-YFP/RFP traced cells predominately expressing the astrocytic marker GFAP within the injured striatal parenchyma after middle cerebral artery occlusion (MCAO) (Li et al., 2010), in the cortex after injury (PBS filling brain cavity) (Wang et al., 2019), cortical stroke (Benner et al., 2013; Faiz et al., 2015) or stab-wound injury (Burns et al., 2009). These studies reveal that the SVZ produces a more predominant astrocytic component than previously appreciated.

These lineage tracing experiments not only provide strong evidence of SVZ-derived astrocytes migrating to sites of injury, but also demonstrate that several types of injury can elicit this response. Additional injuries that also elicit astrocyte differentiation responses from SVZ-NSCs include: motor cortex lesion (Saha et al., 2013), cortical stab wound injury (Givogri et al., 2006), aspiration of the left frontoparietal cerebral (Goings et al., 2004), cortical contusion injury (Radomski et al., 2013), controlled cortical impact injury (Kernie et al., 2001), and puncture wound (Mundim et al., 2019). Interestingly, clonal studies also revealed that astrocytes from the SVZ migrate to the injury site following fine needle injury (Martín-López et al., 2013).

In addition to the cortex, there is evidence that multiple types of SVZ cells migrate to the striatum after injury. SVZ cells migrate toward the striatum in the 6-hydroxydopamine model of Parkinson’s disease, illustrating their migration in neurodegenerative diseases as well as injury. Using Nestin-CreER^T2^, striatal TGFα infusion recruits a large population of SVZ-derived multipotent “C-like” cells to the DA-depleted striatum (De Chevigny et al., 2008). Transient migration of PSA-NCAM/Bromodeoxyuridine (BrdU)-positive cells from the SVZ to the striatum also has been described in the 6-hydroxydopamine implying that newborn cells are capable of migrating into the dopamine deprived striatum (Liu et al., 2006). Another study using BrdU reported that SVZ progenitors proliferate following 6-hydroxydopamine-lesioned striatum after intraventricular injection. Although they report that BrdU + cells co-expressing the astrocytic marker GFAP are widely distributed throughout the lesioned striatum, the authors did not attribute astrogenesis to SVZ progenitors (Aponso et al., 2008).

The type of cells migrating to the injury site can depend on timing post-injury (i.e., short- vs. long- term responses), the type of injury (mechanical vs. chemical), and brain location (proximal or distal from the SVZ). For instance, following stroke or traumatic brain injury (TBI), NPCs migrate toward the injury site to form astrocytes. After these injuries, newly formed neurons are less abundant and do not integrate well, suggesting that NPCs differentiation depends on local environment cues. Additional lineage tracing studies that accounts for timing, location, and cellular diversity (see below) are needed to further investigate how spatial-temporal factors influence SVZ-progenitor fate decisions responses to various forms of injuries. Interestingly, one *in vitro* study showed that murine astrocytes isolated from different brain regions (hippocampus, striatum and cortex) had differential sensitivity to injury (Xu et al., 2001).

It has become increasingly clear that CNS astrocytes represent a diverse cell population (Chai et al., 2017; John Lin et al., 2017; Morel et al., 2017) and it is possible that different classes of astrocytes have distinct functions during tissue recovery after injury. However, whether SVZ-generated astrocytes migrating to the injury site represent a distinct subset of astrocytes remains unclear, and determining if parenchymal astrocytes and SVZ-derived astrocytes have different functions in brain repair is of interest, as it remains poorly defined. Finally, understanding if modulation of these astrocyte populations following injury can stimulate repair may reveal therapeutic targets to improve outcomes after brain injury.

## Molecular Regulators of Subventricular Zone-Derived Astrocytes

Recent studies have defined a molecular framework for developmental astrogenesis that includes the following signaling pathways and transcription factors: Notch, BMP, NFIA, STAT3, Sox9, and LIF (for review Molofsky et al., 2012; Sloan and Barres, 2014; Akdemir et al., 2020). Therefore, in this review, we will discuss the role of these developmental regulators in SVZ- astrogliogenesis with a focus on factors that positively regulate astrogliogenesis in the SVZ in the context of injury (Table 1).

**TABLE 1:** Factors modulating SVZ-astrogliogenesis.

SVZ-Astrogenesis											
**Pathway**	**Factor name**	**Factor impact**			**Role**		**Factor type**		**Factors associated**	**Source**	**References**
		**Proliferation**	**Differentiation**	**Migration**	**Normal**	**Injury**	**Extrinsic**	**Intrinsic**			
NOTCH	Thbs4			x		x	x		Notch/NFIA	SVZ astrocytes	1
	Notch	x	x		x	x	x		NFIA/Thsb4/STAT3	NPC	1–3
	NFIA	x	x		x	x		x	Thbs4	Astrocytes	4
	Sox9	x	x		x	?		x	Notch/NFIA	Astrocytes, SVZ-NSC	5–8
	STAT3		x		x	x		x	Notch/NFIA	SVZ-NSC	3
JAK/STAT	Endothelin-1	x	x			x	x		JAK2/STAT3	Astrocytes	9
	LIF		x		x	?	x		JAK/STAT	*N/A *in vitro treatment*	10,11
BMP	BMP4		x		x	x	x		pSMAD 1/5/8	*N/A *in vitro* treatment	12–14
	ID3		x			x		x	BMP2	NSPC	15
	P57kip2		x		x		x		BMP4, Noggin, Chordin	SVZ Sox2/GFAP + cells	1
	Galectin-3		x		x	x	x		BMP, pSmad1/5/8	SVZ	17
	Fibrinogen		x			x	x		BMP, pSmad1, ID3	*N/A Pharmacological depletion	18

This table summarizes factors modulating SVZ-astrogliogenesis and their effects on proliferation, migration and differentiation according to the following references. Factors are defined as followed: intrinsic factors (transcription factors, receptors) vs. extrinsic factors (growth factors, secreted molecules). 1 Benner et al. (2013); 2 Givogri et al. (2006); 3 Namihira et al. (2009); 4 Laug et al. (2019); 5 Stolt et al. (2003); 6 Cheng et al. (2009); 7 Sun et al. (2017); 8 Kang et al. (2012); 9 Cheng et al. (2019); 10 Bonaguidi et al. (2005); 11 Bauer and Patterson (2006); 12 Gross et al. (1996); 13 Gomes et al. (2003); 14 Cate et al. (2010); 15 Bohrer et al. (2015); 16 Jadasz et al. (2012); 17 Al-Dalahmah et al. (2020); 18 Pous et al. (2020). *The factor source is not indicated N/A as it is applied in vitro or by external administration.

## Notch Pathway

### Thrombospondin 4

Localized photothrombotic/ischemic cortical injury initiates a marked increase in Thrombospondin 4 high (Thbs4hi) astrocyte production from the postnatal SVZ and these cells home to the injured cortex. Thbs4 homozygous knockout mice (Thbs4*^KO/KO^*) demonstrated severe defects in cortical-injury-induced SVZ astrogenesis, resulting in abnormal glial scar formation (Benner et al., 2013). This robust post-injury astrogenic response requires SVZ Notch activation, modulated by Thbs4 via direct Notch1 receptor binding and endocytosis to activate downstream signals, including increased expression of the transcription factor NFIA, which is important for developmental gliogenesis (Deneen et al., 2006). In another report, *Thbs4^KO/KO^* animals exhibited impaired migration of newly formed neurons along the RMS, with several neurons migrating out of the RMS (Girard et al., 2014). Together, these studies suggest a role for Thbs4 associated with Notch and NFIA in SVZ-issued astrocytes migration following injury.

### NFIA

NFIA plays a crucial role in the onset of gliogenesis, astrocyte differentiation, and maintaining morphological integrity of astrocytes in the adult hippocampus (Kang et al., 2012; Huang A. Y.-S. et al., 2020). NFIA is highly expressed in SVZ-NSCs and plays a general role in maintaining proliferative cell populations in the SVZ under homeostatic conditions (Laug et al., 2019). In addition, NFIA is required for SVZ proliferation in the uninjured brain and after cortical ischemia, suggesting that defects in reactive astrogenesis could be a result of these defects in the SVZ. Furthermore, absence of NFIA was associated with a decrease in cellular proliferation. After ischemic stroke, NFIA plays a role in the production of reactive astrocytes from the SVZ and its absence was associated with aberrant glial scar formation, highlighted by increased and prolonged blood serum leakage into the parenchyma. Mechanistically, NFIA directly regulates the expression of Thbs4 in the SVZ, revealing a key transcriptional node that contributes to reactive astrogenesis following cortical injuries (Laug et al., 2019).

### Sox9

The transcription factor Sox9 regulates induction of NFIA and plays a crucial role in the onset of gliogenesis (Stolt et al., 2003; Kang et al., 2012), while activation of Notch1 during neuroectodermal differentiation has been shown to upregulate *Sox9* expression (Martini et al., 2013). Furthermore, in adult astrocytes, Sox9-expression is required to maintain morphological integrity of astrocytes in the olfactory bulb (Ung et al., 2021). Overexpression of Sox9 in the adult SVZ suppresses production of neurons from NSCs, whereas Sox9 knockdown stimulates neurogenesis and inhibits gliogenesis (Cheng et al., 2009) indicating that Sox9 promotes astrogenesis in SVZ-NSC populations. The role of Sox9 in SVZ-NSC astrocyte production after injury remains poorly defined, however, Sox9 is expressed in reactive astrocytes after MCAO injury (Sun et al., 2017) suggesting that it may also have a role in injury associated SVZ-astrogenesis.

### Notch/STAT3

During development, NPCs express Notch ligands and activate Notch signaling in neighboring NPCs, conferring astrocytic differentiation potential through the induction of NFIA. This Notch-NFIA pathway potentiates Stat3-activity and further reinforces the astrocytic differentiation program (Namihira et al., 2009). Following cortical stab wound or stroke, the astrogliogenic response of the SVZ to injury is accompanied by activation of the Notch pathway modulated by Thbs4 (Givogri et al., 2006; Benner et al., 2013). Therefore, NOTCH/STAT3 pathway appears to play a central role in SVZ-induced astrogliogenesis response following injury.

## JAK/STAT Pathway

### Leukemia Inhibitory Factor

Leukemia inhibitory factor (LIF) activates the JAK/STAT pathway, which plays an important role in NSC/NSP differentiation into glia. Indeed, the JAK/STAT pathway promotes astrocyte differentiation during development (Bonni et al., 1997) and is specifically activated in subsets of CNS lesions (Okada et al., 2006). Treatment of cultured embryonic SVZ-NPCs with LIF generates GFAP + cells that have the characteristics typical of adult SVZ and SGZ stem cells/astrocytes (Bonaguidi et al., 2005). In addition, LIF impairs neuroblast formation in the SVZ and stimulates the formation of SVZ astrocytes (Bauer and Patterson, 2006). Therefore, under homeostatic conditions LIF, via the JAK/STAT pathway, can promote SVZ-astrogliogenesis. However, because JAK/STAT signaling promotes astrogliogenesis, coupled with the fact that it is activated following CNS injury, suggests that it may also contribute to SVZ-astrogliogenesis following injury.

### Endothelin-1

Astrocytic endothelin-1 overexpression in mice (GET-1) promotes NSC proliferation and astrocytic differentiation via the Jak2/Stat3 pathway after MCAO in the ipsilateral SVZ (Cheng et al., 2019). Therefore, JAK2/STAT3 pathway appears to play a central role in SVZ-induced astrogliogenesis response following injury.

## Bone Morphogenetic Protein Pathway

### Bone Morphogenetic Protein 4

Bone morphogenetic protein (BMP) signaling promotes the generation of astrocytes from the SVZ (Gross et al., 1996; Gomes et al., 2003). BMP receptor activation inhibits proliferation and suppresses SVZ neurogenesis while promoting gliogenesis (Lim et al., 2000). In the absence of injury, BMP4 promotes astrocytic differentiation of SVZ-NPCs both *in vitro* and *in vivo* (Gross et al., 1996; Bonaguidi et al., 2005). During demyelination BMP4 increased levels are associated with an increase in phosphorylated SMAD 1/5/8. Further, treatment with BMP4 or demyelination increased production of astrocytes within the SVZ compared to naïve mice indicating that BMP4 is sufficient to promote SVZ-produced astrocytes (Cate et al., 2010).

### ID-3, P57kip2, and Agmantine

In addition, genetic depletion of the transcriptional regulator Id3 (BMP-2-induced transcriptional regulator) decreased the number of astrocytes generated from SVZ-derived adult NSCs/NSPs in the cortical lesion area after TBI (Bohrer et al., 2015). Deletion of P57kip2, an important upstream promoter of BMP4-mediated astrogliogenesis, abrogated astrogenesis from SVZ-NSCs, possibly through increased Noggin and chordin levels (Jadasz et al., 2012) comforting the role of the BMP pathway in SVZ-astrocytes production following injury. In SVZ-NSCs cultures, treatment with Agmatine, an endogenous primary amine, increased ERK1/2 expression and suppressed astrogenesis by decreasing expression of BMP 2,4 and SMAD 1,5,8 in SVZ-NSCs (Song et al., 2011) confirming the involvement of BMP and SMAD in SVZ-astrocytes response after injury.

### Galectin-3

In another series of BMP-associated studies, galectin-3 (Gal-3) overexpression increased the percentage of striatal astrocytes generated by the SVZ, coupled with a concomitant decrease in the percentage of oligodendrocytes. Mechanistically, Gal-3 induced BMP signaling by binding to the BMP receptor one alpha (BMPR1α) and by increasing the phosphorylation of pSmad1/5/8, BMP signaling in turn suppressed Gal-3 expression. Indeed, Gal-3 mRNA levels were reduced 24 and 48 h after BMP4 treatment of SVZ cells, suggesting negative feedback of BMP on Gal-3 transcription. In the same study, in human following hypoxia/ischemia, Gal-3 immunoreactivity was increased in the perinatal human SVZ and striatum suggesting a role of Gal-3 in promoting SVZ-gliogenesis after injury (Al-Dalahmah et al., 2020). Of note, Gal-3 appears to play an important role in non-SVZ gliogenesis as well, as its deletion led to a reduction in the number of striatal glial populations, whereas its overexpression led to an increase in glial production.

### Fibrinogen

In addition, fibrinogen which is enriched in the SVZ niche following cortical brain injury in mice, inhibited neuronal differentiation in SVZ and hippocampal NSPs/NSCs while promoting astrogenesis via activation of the BMP receptor signaling pathway. These results suggest that fibrinogen is a regulator of NSPC-derived astrogenesis from the SVZ niche via BMP receptor signaling pathway following injury (Pous et al., 2020). Collectively, these studies demonstrate that activation of BMP signaling promotes SVZ-astrogliogenesis under both homeostatic and injury conditions.

**FIGURE 2:**
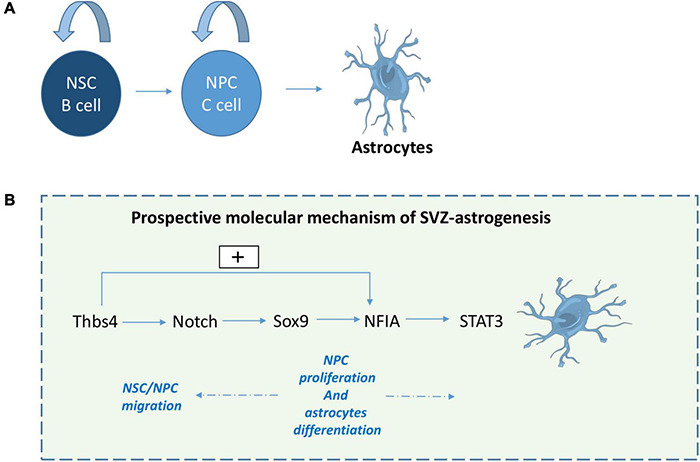
Prospective molecular mechanism of SVZ-derived astrogliogenesis. Following stroke or TBI, NPCs give rise to astrocytes **(A)**. Here we suggest a prospective mechanism for SVZ astrogenesis **(B)**: 1- Thbs4 activates downstream effectors of the Notch pathway and NFIA, 2- Sox9 regulates NFIA induction, while Notch activation upregulates Sox9 expression, 3- NFIA directly regulates Thbs4-expression and 4- Notch activates NFIA leading to STAT3-activating signal which play a role in NPC proliferation and astrocytes differentiation.

To conclude, several pathways can influence SVZ-astrogliogenesis. Taking into account the relationship between these factors, we propose a potential pathway between Thbs4, Notch, Sox9, NFIA and Stat 3 that drives SVZ-astrogenesis. It begins with Thbs4 physically interacting with Notch1 to activate downstream effectors of the Notch pathway (Givogri et al., 2006; Benner et al., 2013) and inducing NFIA expression in primary SVZ-NSCs (Benner et al., 2013). Next, Sox9 contributes to the induction of NFIA expression (Kang et al., 2012), while activation of Notch1 upregulates Sox9 expression, thus reinforcing the transcriptional apparatus that confers astrocyte identity (Martini et al., 2013). Subsequently, NFIA directly regulates Thbs4-expression (Laug et al., 2019) and Notch activates NFIA leading to STAT3-activating signals (Namihira et al., 2009) providing a feed-forward, self-propagating gliogenic signaling mechanism (Figure 2).

## The Subventricular Zone Produces Myelinated Oligodendrocytes Following Demyelination

SVZ-NSCs are also able to produce oligodendrocytes as discussed above. Parenchymal oligodendrocyte precursor cells (OPCs) and SVZ-derived progenitors are the two main sources of progenitor cells that contribute to oligodendrogenesis (for review El Waly et al., 2014). A small fraction of SVZ-NSCs are also able to generate OPCs that migrate out of the SVZ into the overlying white matter and cortex (Menn et al., 2006).

The number of oligodendrocytes derived from NSCs increased fourfold after a demyelinating lesion in the CC, indicating that SVZ-NPCs participate in myelin repair in the adult brain following injury (Nait-Oumesmar et al., 1999; Picard-Riera et al., 2002; Menn et al., 2006). Examination of post-mortem human Multiple Sclerosis (MS) brains revealed the migration of SVZ-OPCs to periventricular lesions, where they could participate in remyelination (Nait-Oumesmar et al., 2007). In mice, SVZ-OPCs migrate during the remyelination phase after cuprizone-induced demyelination to the CC and are capable of forming new oligodendrocytes (Butti et al., 2019), while also producing thicker myelin sheets (Xing et al., 2014). Therefore, stimulating the regenerative potential of SVZ-NPCs could be a promising strategy for therapies for demyelinating diseases such as MS.

In the nestin-CreERT2-R26R-YFP mouse model, demyelination led to decreased neurogenesis in the adult brain (Luo et al., 2020) that was coupled with a massive recruitment of SVZ-NSCs to the demyelinated CC during the acute phases of injury response, where these recruited cells subsequently differentiated into myelinating oligodendrocytes (Brousse et al., 2015). In addition, ablation of SVZ-NSCs using Ganciclovir during cuprizone-induced demyelination resulted in reduced numbers of oligodendrocytes within the lesioned CC (Butti et al., 2019), while local irradiation of the SVZ preserves the capacity of NSCs to respond to a demyelinating lesion in the striatum and differentiate in oligodendrocytes (Capilla-Gonzalez et al., 2014).

Following experimental autoimmune encephalomyelitis (EAE) (Calzà et al., 1998; Picard-Riera et al., 2002), aspiration induced cortical lesions (Goings et al., 2004), or traumatic axonal injury (Sullivan et al., 2013), SVZ cells also migrate from the SVZ to the CC and differentiate into oligodendrocytes. Hypoxic-ischemic insult also stimulates SVZ-NSCs to generate new neurons and oligodendrocytes *in vitro* (Yang and Levison, 2006) or *in vivo* (Zaidi et al., 2004). OPC expression of NG2-/Olig2 in the SVZ occurs within the first few days after hypoxia (Jablonska et al., 2012), with one study reporting an increase in the number of Olig2 + cells in the posterior part of the SVZ, which subsequently migrated into the injured white matter (Kako et al., 2012).

In a model of demyelination following injection of lysolecythin in the anterior CC, SVZ-NPCs labeled by direct injection of cytomegalovirus (CMV)-GFP retrovirus revealed co-labeling with Olig2 and the proteoglycan NG2. These cells were predominately located within the CC. In addition, spontaneous excitatory postsynaptic currents significantly increased 1 week after the lesion, indicating that oligodendrocytes became synaptically connected (Etxeberria et al., 2010). An additional study used genetic fate mapping following acute demyelination by local injection of α-lysophosphatidylcholine (LPC) in the CC to demonstrate that local OPCs rapidly respond and expand in the lesion within 7 days, and produced oligodendrocytes within 2 weeks after injury. By contrast, NSC-derived NG2 cells did not significantly increase in the lesion until 4 weeks after demyelination and generated fewer oligodendrocytes than parenchymal OPCs.

These observations suggest that local OPCs may be the primary responders to repair acutely demyelinated lesion whereas SVZ-NSCs may contribute to repopulating OPCs following their depletion due to oligodendrocyte differentiation (Serwanski et al., 2018).

In contrast, another study reported that SVZ-derived cells of the oligodendroglial lineage that migrate either to the intact or the focally demyelinated CC have limited migratory and self-renewal capacity and fail to generate mature myelin (Kazanis et al., 2017). A further study proposed that SVZ-NSCs are dispensable for myelin repair but protect neurons from degeneration (Butti et al., 2019). Beyond their role replacing oligodendrocytes, SVZ-NSCs also display immunomodulatory properties, highlighting a new role for endogenous SVZ-NSC in myelin regeneration. Indeed, SVZ-NSC may minimize demyelination by modulating microglial activity and promoting myelin debris phagocytosis (Brousse et al., 2020).

## Molecular Regulators of Subventricular Zone-Derived Oligodendrocytes

Recently, several studies have demonstrated that a range of factors such as LIF/CNTF, Endothelin-1, Notch, EGFR play a major role in oligodendrogenesis (for review Gonzalez-Perez and Alvarez-Buylla, 2011; Maki et al., 2013; El Waly et al., 2014; Adams et al., 2020). In this review, we will summarize the role of these factors in SVZ-oligodendrogenesis with a focus on factors positively regulating oligodendrogenesis in the SVZ in the context of injury and especially white matter injury (Table 2).

**TABLE 2:** Factors modulating SVZ-oligodendrogenesis.

SVZ-Oligodendrogenesis													
**Pathway**	**Factor name**	**Factor impact**					**Role**		**Factor type**		**Factors associated**	**Source**	**References**
		**Proliferation**	**Differentiation**	**Migration**	**Maturation**	**Other**	**Normal**	**Injury**	**Extrinsic**	**Intrinsic**			
Notch	Endothelin-1	x	x	x			x	x	x		EDNRB, Notch, Jagged 1. Gsx1, S100b	Endothelial cells/Astrocytes	1–3
	Jagged 1	x						x	x		NICD, HES5	Reactive astrocytes SVZ, CC	4
	TGF-β		x				x		x		Jagged1, HES1	*N/A *in vitro* treatment	5
	F3/Contactin				x			x	x		Notch, Apotransferrin	Neurons	4
	Apotransferrin	x	x					x	x		Notch	*N/A Intranasal treatment	6
BMP	Noggin	x	x				x	x	x		BMP4, pSMAD1/5/8	SVZ-NSC	7,8
JAK/STAT	CNTF			x			x	x	x		JAK/STAT	Astrocytes, SVZ, lesion site	9
	LIF	x	?				x	?	x		JAK/STAT	LV administration	10
Wnt	Canonical Wnt		x				x		x			SEZ	11
	SFRP1 and SFRP5		x					x	x		Wnt, BMAL1	Astrocytes in demyelinating lesions	12
Growth factors	FGF receptor-3		x					x		x	FGF	SVZ-NCS	13
	Anosmin-1	x		x			x		x		FGFR1 receptor	SVZ-NP, Astrocytes	14
	EGF, FGF-2, and PDGF		x	x			x		X			*N/A intraperitoneal injection	15
	EGF			x				x	X			*N/A infusion lateral ventricle	16
					x			x	X			* N/A intranasal administration	17
	HB-EGF			x				x	X			*N/A intranasal administration	18
	EGFR	x	x					x		x		SVZ/CC	19
						regeneration		x		x		oligodendrocytes lineage	17
	N-cadherin			x				x	X		EGFR, ADAM10	SVZ NPCs (EGFR + cells)	20
	IGF-1					myelination and protection		x	x			*N/A subcutaneous/intraventricular injection	21,22
	T3 free window	x					x	x	x		EGFR	*N/A Food treatment	23
SHH	SmoM2		x				x	?	x			DV-SVZ	24
Others	Cdk4	x						x	x			SVZ	25
	Prickle1		x		x		x		x			NSCs, OPcs	26
	Nfe2l3		x		x		x		X			NSCs, OPcs	26
	Asialo-erythropoietin		x		x			x	X			*N/A intraperitoneal injection	27
	Zfp488		x		x			x	X			SVZ NSCs	28
	FTY720	x	x				x	x	X		Src-bcr-Abl tyrosine kinase, S1P receptor	*N/A in vitro treatment, intraperitoneal injection	29

This table summarizes factors modulating SVZ-oligodendrogenesis and their effects on proliferation, migration, differentiation according to the following references. Factors are defined as followed: intrinsic factors (transcription factors, receptors) vs. extrinsic factors (growth factors, secreted molecules). 1 Gadea et al. (2009); 2 Hammond et al. (2015); 3 Adams et al. (2020); 4 Aparicio et al. (2013); 5 Pinto et al. (2018); 6 Guardia Clausi et al. (2012); 7 Morell et al. (2015); 8 Cate et al. (2010); 9 Vernerey et al. (2013); 10 Bauer and Patterson (2006); 11 Ortega et al. (2013); 12 Huang S. et al., 2020; 13 Kang et al. (2019); 14 Murcia-Belmonte et al. (2016); 15 Lachapelle et al. (2002); 16 Gonzalez-Perez et al. (2009); 17 Scafidi et al. (2014); 18 Cantarella et al. (2008); 19 Aguirre et al. (2007); 20 Klingener et al. (2014); 21 Lin et al. (2005); 22 Zhong et al. (2009); 23 Remaud et al. (2017); 24 Tong et al. (2015); 25 Jablonska et al. (2012); 26 Zilkha-Falb et al. (2017); 27 Kako et al. (2012); 28 Soundarapandian et al. (2011); 29 Cipriani et al. (2017). *The factor source is not indicated N/A as it is applied in vitro or by external administration.

## Notch Pathway

### Endothelin-1

Endothelial cells produce endothelin (ET-1), which promotes oligodendrocyte differentiation in the SVZ. ET-1 stimulates a pro-migratory phenotype in cultured OPCs and SVZ explants, while selective ET receptor antagonists or anti-ET-1 antibodies inhibit OPC migration from the SVZ (Gadea et al., 2009). Another study revealed that ET-1 acts selectively through EDNRB on astrocytes, but not OPCs, to indirectly inhibit remyelination (Hammond et al., 2015). Loss of ET-1 signaling increases neurogenesis and reduces OPC in the developing SVZ. In addition, ET-1 acts mechanistically by promoting Notch activation in OPCs during remyelination through induction of Jagged1 expression in reactive astrocytes (Hammond et al., 2015; Adams et al., 2020). ET-1 also induces upregulation of *Gsx1* and downregulation of *S100b* in SVZ OPCs, which acts to increase their proliferation as well (Adams et al., 2020). Together, these studies reinforce the notion that ET-1 is required for increased NPCs and OPC proliferation in the adult mouse SVZ following demyelination (Adams et al., 2020).

### Jagged 1, TGF-β, F3/Contactin, Apotransferrin

Levels of Notch ligand Jagged1 increase in the CC and SVZ during the early phases of LPC-mediated demyelination in rats. This increase was found to induce Notch intracellular domain (NICD) release and Hes5 expression, which increased OPC proliferation (Aparicio et al., 2013). TGF-β also has pro-oligodendrogenic effects on adult SVZ progenitors *in vitro* and induced the expression of Jagged1 and downstream gene Hes1 (Pinto et al., 2018). In this context it appears that Notch activation is mediated by the expression of F3/contactin, which could then induce apotransferrin-mediated oligodendroglial maturation (Aparicio et al., 2013). Indeed, intranasal administration of apotransferrin enhanced OPC proliferation in the SVZ and CC and promoted OPC differentiation (Guardia Clausi et al., 2012). Therefore, these studies suggest a role for Notch involving apotransferrin in promoting OPC proliferation and oligodendrocytes differentiation.

## Bone Morphogenetic Protein Pathway

### Noggin

Noggin promotes the proliferation of SVZ-NSCs, and shifts the differentiation of NSCs from mature astrocytes to transit amplifying NPCs and OPCs without depleting the NSC population (Morell et al., 2015). Intraventricular infusion of Noggin, which is an endogenous antagonist of BMP4, reduced pSMAD1/5/8, decreased astrocyte numbers, and increased oligodendrocyte numbers in the SVZ during cuprizone-induced demyelination (Cate et al., 2010). Therefore, these results suggest that Noggin promotes oligodendrogenesis following demyelination injury.

## JAK/STAT Pathway

### Leukemia Inhibitory Factor/Ciliary Neurotrophic Factor

Ciliary neurotrophic factor (CNTF) and LIF are neurotrophic cytokine belonging to the interleukin-6 (IL6) family that activates the JAK/STAT pathway, which also plays an active role in astrogenesis.

LIF stimulates the self-renewal of adult NSCs in the SVZ, which may expand this population to facilitate repair (Bauer and Patterson, 2006). This finding has relevance for the repair of demyelination since NSCs can generate migratory OPCs that differentiate into oligodendrocytes and contribute to remyelination (Menn et al., 2006).

On the other hand, CNTF (Ciliary neurotrophic factor) controls the migration of SVZ-derived progenitors following HEK cells secreting CNTF graft into the CC and also controls OPCs toward the demyelinated CC both *in vivo* and in *in vitro* models (Vernerey et al., 2013) suggesting a role for both LIF and CNTF in oligodendrocytes migration/differentiation following demyelination.

## WNT Pathway

### Canonical Wnt

Shifts between non-canonical and canonical Wnt signaling activate quiescent NSCs during demyelination injury (Chavali et al., 2018). *In vivo* activation or inhibition of canonical Wnt signaling increased or decreased the number of Olig2 and PDGFR-α positive cells, respectively, suggesting that this pathway contributes to the fine tuning of oligodendrogliogenesis in the adult SVZ (Ortega et al., 2013).

### SFRP1 and SFRP5

Evidence suggests that altered-clock-derived signals in the demyelinated lesion mediate communication with the SVZ to switch NSCs toward generation of oligodendrocyte lineage cells, which enhances remyelination. Astrocyte circadian clocks produce the Wnt inhibitors SFRP1 and SFRP5 that signal to the SVZ to reduce the circadian transcription factor BMAL1 (Huang S. et al., 2020). Together, these studies indicate that Wnt signaling promotes oligodendrogenesis issued from the SVZ in the demyelination lesion.

## Growth Factors

### Epidermal Growth Factor and Fibroblast Growth Factor Signaling

Epidermal growth factor (EGF) and fibroblast growth factor (FGF) signaling pathways play key roles in a spectrum of developmental and physiological processes, including SVZ-NSC responses. Increasing FGF receptor-3 activity in adult SVZ-NSCs cells in a *Nestin-CreER* mouse model transiently promoted differentiation from the neuronal to the oligodendroglial lineage following demyelination and improved myelin repair in the CC and in the lower cortical layers (Kang et al., 2019). In addition, anosmin-1 over-expression was shown to regulate OPCs proliferation, migration and myelin sheath thickness predominately through FGFR1 receptor (Murcia-Belmonte et al., 2016).

EGFR-dependent, N-cadherin signaling promotes migration of oligodendrocytes or oligodendrocyte progenitors into demyelinated lesions (Klingener et al., 2014). Additional studies also demonstrated that NG2^+^ cells responses in the SVZ and differentiation in CC after focal demyelination are dependent upon EGFR signaling (Aguirre and Gallo, 2007). EGFR overexpression in the SVZ and CC during early postnatal development expanded oligodendrocytes progenitors, which enhanced the generation of oligodendrocytes and subsequent axonal myelination in the lesion (Aguirre et al., 2007). Together, these studies indicate that EGF signaling plays a role in SVZ-NSC responses following demyelination injury by promoting oligodendrogenesis. Taken further, direct administration via intraperitoneal injection of EGF, FGF-2, and platelet-derived growth factor (PDGF) promoted the survival, migration, and differentiation of grafted SVZ cells into myelin-forming oligodendrocytes. This combination of growth factors expanded the constitutively proliferative PSA-NCAM + population *in vivo* and facilitated their differentiation toward the neuronal and oligodendroglial cell fates (Lachapelle et al., 2002). Furthermore, EGF infusion into the lateral ventricle (Gonzalez-Perez et al., 2009) or intranasal HB-EGF administration (Cantarella et al., 2008) promoted OPC recruitment from the SVZ to demyelinated lesions.

Importantly, endogenous EGF is upregulated in the white matter and SVZ after perinatal hypoxia and EGFR overexpression in the oligodendrocyte lineage enhances their regeneration and promotes functional recovery in white matter (Scafidi et al., 2014). In addition, in the SVZ, Notch regulates NSC identity and self-renewal, whereas EGFR specifically affects NPC proliferation and migration. Enhanced EGFR signaling resulted in the expansion of the NPC pool and reduced NSC number and self-renewal by inhibiting Notch signaling (Aguirre et al., 2010). Intranasal EGF was also shown to accelerate oligodendrocyte maturation in white matter after chronic neonatal hypoxia (Scafidi et al., 2014) suggesting a role of growth factors in promoting SVZ-issued oligodendrogenesis.

### Insulin-Like Growth Factor I

It was also shown that administration of a related-growth factor, Insulin-like growth factor I (IGF-1), prevented immature oligodendrocyte death, enhanced myelination after hypoxia/ischemia and protected OPCs in the SVZ and white matter regions (Lin et al., 2005; Zhong et al., 2009) suggesting that additional IGF-1 associated pathway promote SVZ-issued oligodendrogenesis.

### Thyroid Hormone (TH) Signaling—T3

EGFR^+^ oligodendrocyte progenitors, but not neuroblasts, express high levels of a T_3_-inactivating deiodinase, Dio3. T_3_ through its nuclear receptor, TRα1, favors progenitor commitment toward a neuroblast phenotype. However, a transient T_3_-free window increases OPCs numbers. Therefore T3 free window provides a favorable environment for SVZ-derived oligodendrocyte progenitor generation (Remaud et al., 2017). This finding indicates that T3 free window promotes remyelination and therefore, plays a role in SVZ-issued oligodendrocytes.

## Sonic Hedgehog Pathway

### SmoM2

Smoothened (Smo) is essential for Sonic hedgehog signaling. Genetic ablation of Smo in the dorsal SVZ resulted in a reduction of oligodendroglial cells in the CC. In contrast, expression of constitutively active SmoM2 significantly increased the number of oligodendrocytes (Tong et al., 2015). Overall, these results suggest that Smo increases SVZ-issued oligodendrocytes and suggest a potential role for Smo in demyelination injury, though these mechanisms have not been directly examined in this context.

## Others Associated Pathways

### CDK4, Prickle1, and Nfe2l3

Hypoxia-induced proliferation of NG2-/Olig2-expressing OPCs occurs in the SVZ within the first few days after insult and depends on activation of the Cdk4 pathway (Jablonska et al., 2012). Subsequent studies demonstrated that Prickle1 and Nfe2l3 are strongly associated with differentiation toward myelin-producing cells. Prickle1 dramatically affects OPCs maturation and differentiation to mature myelinating oligodendrocytes, while Nfe2l3 has a moderate effect on OPC maturation in the absence of injury (Zilkha-Falb et al., 2017). Overall, these studies suggest a role of Cdk4 and Prickle1 in SVZ-induced oligodendrogenesis with a potential role in the context of injury.

### Asialo-Erythropoietin, Zfp488, FTY720

Long-term post-injury treatment with a non-erythropoietic derivative of EPO, asialo-erythropoietin, promoted the maturation of the posterior SVZ-derived OPCs and the recovery of neurological function, without affecting hematopoiesis (Kako et al., 2012). On the other hand, overexpression of oligodendrocyte-specific zinc finger transcription repressor (Zfp488) retrovirus in SVZ-NSCs following Cuprizone-induced demyelination in mice promoted differentiation into mature oligodendrocytes (Soundarapandian et al., 2011). In addition, FTY720, a Src-bcr-Abl tyrosine kinase inhibitor and S1P receptor agonist, increased postnatal SVZ-NSCs differentiation into both neurons and oligodendrocytes *in vitro* and partially increased proliferation and differentiation of OPC after kainic acid lesion *in vivo* (Cipriani et al., 2017).

**FIGURE 3:**
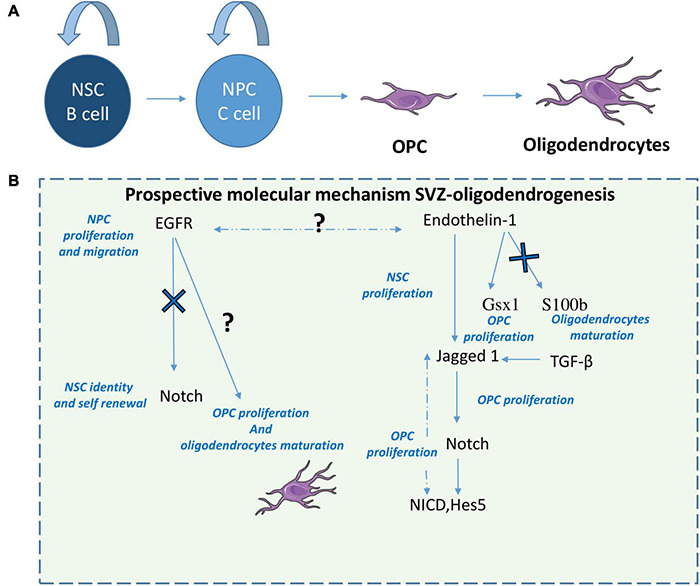
Prospective molecular mechanisms of SVZ-derived oligodendrogenesis. After demyelination or other white matter injuries, NPCs give rise to OPCs that will differentiate in oligodendrocytes **(A)**. Here we suggest a prospective mechanism that modulates SVZ oligodendrogenesis **(B)**. 1-EGFR signaling increases NPC proliferation and migration but also oligodendrocyte maturation by inhibiting Notch. 2-ET-1 promotes Notch activation in OPCs during remyelination through induction of Jagged1 and increases NPCs and OPC proliferation. 3- ET-1 also induces upregulation of *Gsx1* and downregulation of *S100b* in SVZ OPCs, increasing their proliferation but blocking oligodendrocytes maturation. 4-TGF-β has pro-oligodendrogenic effects by increasing Jagged 1.

These collective studies implicate a role for SVZ-NSC derived oligodendrocytes in myelin-associated injury responses and highlight several pathways and strategies that are able to promote SVZ-oligodendrogenesis and myelin repair following demyelination. It appears that Notch has a dual role in oligodendrogenesis being both inhibited by EGFR signaling which promotes OPC proliferation and oligodendrocytes differentiation (Aguirre et al., 2010; Scafidi et al., 2014) but also being activated by Endothelin-1 and Jagged 1 [also activated by TGF B (Pinto et al., 2018)] to promote OPC proliferation through NICD and Hes5 (Adams et al., 2020). Interestingly Endothelin-1 also plays a role on OPC proliferation by activation Gsx1 but also inhibits oligodendrocytes maturation through S100b inhibition (Adams et al., 2020; Figure 3). Taking this dual role of Notch, it would be of interest following demyelination to assess EGFR signaling and Endothelin-1 signaling relationship.

## Conclusion

In conclusion, astrocytes and oligodendrocytes play pivotal roles in diverse injury responses throughout the CNS and the type of injury and location in the brain where the injury occurs dictates their generation by SVZ-NSCs. Indeed, ischemia and TBI induce the SVZ to produce astrocytes, whereas oligodendrocytes are produced after white matter injury and demyelinating events. Furthermore, the fate of SVZ-NSCs under physiological conditions is regulated by the combined actions of intrinsic and extrinsic factors, in addition of regional differences within the SVZ. Although several key features of these two cell types under normal physiological conditions and in response to injury have emerged in recent decades, additional studies combining new genetic tools, molecular studies, and behavioral tests are warranted to further decipher how these injury-specific responses regulate SVZ production of astrocytes or oligodendrocytes.

In addition, SVZ-issued astrocytes play a major role in glial scar formation. It is unclear whether SVZ-generated astrocytes migrating to the injury site, 1- represent a distinct subset of astrocytes compared to parenchymal astrocytes, 2- have different functions in brain repair and capacity to integrate to the existing brain circuitry. Furthermore, future studies should consider SVZ-astrocytes as a strategy to compensate for neuronal loss following injury. The adult brain cortex has limited ability to produce new neurons, therefore reprogramming astrocytes into neurons could be an ideal approach to replenish the lost cells and repair the damage. As it can be challenging to reprogram fully differentiated astrocytes and that NSCs make newly formed astrocytes contributing to the glial scar following injury, there is a potential for *in vivo* reprogramming of SVZ-issued astrocytes into neurons.

SVZ-issued oligodendrocytes also play a major role in re-myelination and their proliferation rate or subtype may also differ from parenchymal oligodendrocytes. SVZ-OPCs are recruited during the remyelination phase to the CC and are capable of forming new oligodendrocytes. Therefore, future studies should focus on promoting SVZ-OPCs proliferation and differentiation by endogenous or exogenous factors in order to promote myelin repair. It would also be of interest to establish therapies stimulating myelin repair to prevent neurodegeneration in pathology like MS.

Lastly, there are sub-regional differences of the SVZ niche with respect to embryonic origins and cell subtype generation (Young et al., 2007), where single-cell analysis revealed that SVZ lateral and septal wall astrocytes are primarily neurogenic and oligodendrogenic, respectively (Mizrak et al., 2019). This suggests functionally relevant spatial diversity in neurogenesis and oligodendrogenesis in the adult brain, while also revealing molecular correlates of adult NSC dormancy and lineage specialization (Mizrak et al., 2019). To our knowledge, single-cell analysis on SVZ subregions involved in astrogenesis has yet to be performed and could provide insight into the origins of diverse astrocyte responses. Therefore, future studies should assess astrogliogenesis modulation by specific factors in specific SVZ subregions in order to understand specific sub-SVZ regions implicated in SVZ-astrogliogenesis response following injury. Besides, the dorsal SVZ appears to be more oligodendrogliogenic than the lateral SVZ, consistent with the notion of a mosaic organization of the SVZ (Merkle et al., 2007; Cebrian-Silla et al., 2021; Delgado et al., 2021). Therefore, future studies assessing oligodendrogliogenic modulation by specific factors in specific SVZ subregions are necessary to increase our understanding of specific sub-SVZ regions implication in SVZ-oligodendrogenesis response following injury.

## Author Contributions

All authors listed have made a substantial, direct, and intellectual contribution to the work, and approved it for publication.

## Conflict of Interest

The authors declare that the research was conducted in the absence of any commercial or financial relationships that could be construed as a potential conflict of interest.

## Publisher’s Note

All claims expressed in this article are solely those of the authors and do not necessarily represent those of their affiliated organizations, or those of the publisher, the editors and the reviewers. Any product that may be evaluated in this article, or claim that may be made by its manufacturer, is not guaranteed or endorsed by the publisher.

## References

[B1] Adams K. L. Riparini G. Banerjee P. Breur M. Bugiani M. Gallo V. (2020). Endothelin-1 signaling maintains glial progenitor proliferation in the postnatal subventricular zone. *Nat. Commun.* 11:2138. 10.1038/s41467-020-16028-8 32358570 PMC7195367

[B2] Aguirre A. Dupree J. L. Mangin J. M. Gallo V. (2007). A functional role for EGFR signaling in myelination and remyelination. *Nat. Neurosci.* 10, 990–1002. 10.1038/nn1938 17618276

[B3] Aguirre A. Gallo V. (2007). Reduced EGFR signaling in adult progenitors of the subventricular zone attenuates oligodendrogenesis after demyelination. *Neuron Glia Biol.* 3, 209–220. 10.1017/S1740925X08000082 18634612 PMC2696258

[B4] Aguirre A. Rubio M. E. Gallo V. (2010). Notch and EGFR pathway interaction regulates neural stem cell number and self-renewal. *Nature* 467 323–327. 10.1038/nature09347 20844536 PMC2941915

[B5] Akdemir E. S. Huang A. Y.-S. Deneen B. (2020). Astrocytogenesis: where, when, and how. *F1000Research* 9:F1000 Faculty Rev-233. 10.12688/f1000research.22405.1 32269761 PMC7122459

[B6] Al-Dalahmah O. Campos Soares L. Nicholson J. Draijer S. Mundim M. Lu V. M. (2020). Galectin-3 modulates postnatal subventricular zone gliogenesis. *Glia* 68 435–450. 10.1002/glia.23730 31626379 PMC6916335

[B7] Altman J. (1962). Are new neurons formed in the brains of adult mammals? *Science* 135 1127–1128. 10.1126/science.135.3509.1127 13860748

[B8] Aparicio E. Mathieu P. Pereira Luppi M. Almeira Gubiani M. F. Adamo A. M. (2013). The Notch signaling pathway: its role in focal CNS demyelination and apotransferrin-induced remyelination. *J. Neurochem.* 127 819–836. 10.1111/jnc.12440 24032544

[B9] Aponso P. M. Faull R. L. M. Connor B. (2008). Increased progenitor cell proliferation and astrogenesis in the partial progressive 6-hydroxydopamine model of Parkinson’s disease. *Neuroscience* 151 1142–1153. 10.1016/j.neuroscience.2007.11.036 18201835

[B10] Bauer S. Patterson P. H. (2006). Leukemia inhibitory factor promotes neural stem cell self-renewal in the adult brain. *J. Neurosci.* 26 12089–12099. 10.1523/JNEUROSCI.3047-06.2006 17108182 PMC6674883

[B11] Benner E. J. Luciano D. Jo R. Abdi K. Paez-Gonzalez P. Sheng H. (2013). Protective astrogenesis from the SVZ niche after injury is controlled by Notch modulator Thbs4. *Nature* 497 369–373. 10.1038/nature12069 23615612 PMC3667629

[B12] Bohrer C. Pfurr S. Mammadzada K. Schildge S. Plappert L. Hils M. (2015). The balance of Id3 and E47 determines neural stem/precursor cell differentiation into astrocytes. *EMBO J.* 34 2804–2819. 10.15252/embj.201591118 26438726 PMC4682650

[B13] Bonaguidi M. A. McGuire T. Hu M. Kan L. Samanta J. Kessler J. A. (2005). LIF and BMP signaling generate separate and discrete types of GFAP-expressing cells. *Development* 132 5503–5514. 10.1242/dev.02166 16314487

[B14] Bonaguidi M. A. Song J. Ming G. Song H. (2012). A unifying hypothesis on mammalian neural stem cell properties in the adult hippocampus. *Curr. Opin. Neurobiol.* 22 754–761. 10.1016/j.conb.2012.03.013 22503352 PMC3415562

[B15] Bonni A. Sun Y. Nadal-Vicens M. Bhatt A. Frank D. A. Rozovsky I. (1997). Regulation of gliogenesis in the central nervous system by the JAK-STAT signaling pathway. *Science* 278 477–483. 10.1126/science.278.5337.477 9334309

[B16] Brousse B. Magalon K. Daian F. Durbec P. Cayre M. (2020). Endogenous neural stem cells modulate microglia and protect from demyelination. *bioRxiv* [Preprint]. 10.1101/2020.06.18.158782 PMC8282429 34087164

[B17] Brousse B. Magalon K. Durbec P. Cayre M. (2015). Region and dynamic specificities of adult neural stem cells and oligodendrocyte precursors in myelin regeneration in the mouse brain. *Biol. Open* 4 980–992. 10.1242/bio.012773 26142314 PMC4542288

[B18] Burda J. E. Bernstein A. M. Sofroniew M. V. (2016). Astrocyte roles in traumatic brain injury. *Exp. Neurol.* 275(Pt 3), 305–315. 10.1016/j.expneurol.2015.03.020 25828533 PMC4586307

[B19] Burns K. A. Murphy B. Danzer S. C. Kuan C.-Y. (2009). Developmental and post-injury cortical gliogenesis: a genetic fate-mapping study with nestin-CreER mice. *Glia* 57 1115–1129. 10.1002/glia.20835 19115384 PMC4286201

[B20] Butti E. Bacigaluppi M. Chaabane L. Ruffini F. Brambilla E. Berera G. (2019). Neural stem cells of the subventricular zone contribute to neuroprotection of the corpus callosum after cuprizone-induced demyelination. *J. Neurosci. Off. J. Soc. Neurosci.* 39 5481–5492. 10.1523/JNEUROSCI.0227-18.2019 31138656 PMC6616285

[B21] Calzà L. Giardino L. Pozza M. Bettelli C. Micera A. Aloe L. (1998). Proliferation and phenotype regulation in the subventricular zone during experimental allergic encephalomyelitis: *In vivo* evidence of a role for nerve growth factor. *Proc. Natl. Acad. Sci. U.S.A.* 95 3209–3214. 10.1073/pnas.95.6.3209 9501242 PMC19721

[B22] Cantarella C. Cayre M. Magalon K. Durbec P. (2008). Intranasal HB-EGF administration favors adult SVZ cell mobilization to demyelinated lesions in mouse corpus callosum. *Dev. Neurobiol.* 68 223–236. 10.1002/dneu.20588 18000828

[B23] Capilla-Gonzalez V. Guerrero-Cazares H. Bonsu J. M. Gonzalez-Perez O. Achanta P. Wong J. (2014). The subventricular zone is able to respond to a demyelinating lesion after localized radiation. *Stem Cells Dayt. Ohio* 32 59–69. 10.1002/stem.1519 24038623 PMC4879590

[B24] Cate H. S. Sabo J. K. Merlo D. Kemper D. Aumann T. D. Robinson J. (2010). Modulation of bone morphogenic protein signalling alters numbers of astrocytes and oligodendroglia in the subventricular zone during cuprizone-induced demyelination. *J. Neurochem.* 115 11–22. 10.1111/j.1471-4159.2010.06660.x 20193041

[B25] Cebrian-Silla A. Nascimento M. A. Redmond S. A. Mansky B. Wu D. Obernier K. (2021). Single-cell analysis of the ventricular-subventricular zone reveals signatures of dorsal and ventral adult neurogenesis. *eLife* 10:e67436. 10.7554/eLife.67436 34259628 PMC8443251

[B26] Chai H. Diaz-Castro B. Shigetomi E. Monte E. Octeau J. C. Yu X. (2017). Neural circuit-specialized astrocytes: transcriptomic, proteomic, morphological, and functional evidence. *Neuron* 95 531.e9–549.e9. 10.1016/j.neuron.2017.06.029 28712653 PMC5811312

[B27] Chang E. H. Adorjan I. Mundim M. V. Sun B. Dizon M. L. V. Szele F. G. (2016). Traumatic brain injury activation of the adult subventricular zone neurogenic niche. *Front. Neurosci.* 10:332. 10.3389/fnins.2016.00332 27531972 PMC4969304

[B28] Chavali M. Klingener M. Kokkosis A. G. Garkun Y. Felong S. Maffei A. (2018). Non-canonical Wnt signaling regulates neural stem cell quiescence during homeostasis and after demyelination. *Nat. Commun.* 9:36. 10.1038/s41467-017-02440-0 29296000 PMC5750230

[B29] Cheng L.-C. Pastrana E. Tavazoie M. Doetsch F. (2009). miR-124 regulates adult neurogenesis in the SVZ stem cell niche. *Nat. Neurosci.* 12 399–408. 10.1038/nn.2294 19287386 PMC2766245

[B30] Cheng X. Yeung P. K. K. Zhong K. Zilundu P. L. M. Zhou L. Chung S. K. (2019). Astrocytic endothelin-1 overexpression promotes neural progenitor cells proliferation and differentiation into astrocytes via the Jak2/Stat3 pathway after stroke. *J. Neuroinflammation* 16:227. 10.1186/s12974-019-1597-y 31733648 PMC6858703

[B31] Cipriani R. Chara J. C. Rodríguez-Antigüedad A. Matute C. (2017). Effects of FTY720 on brain neurogenic niches in vitro and after kainic acid-induced injury. *J. Neuroinflammation* 14:147. 10.1186/s12974-017-0922-6 28738875 PMC5525223

[B32] De Chevigny A. Cooper O. Vinuela A. Reske-Nielsen C. Lagace D. C. Eisch A. J. (2008). Fate mapping and lineage analyses demonstrate the production of a large number of striatal neuroblasts after TGFα and noggin striatal infusions into the dopamine-depleted striatum. *Stem Cells Dayt. Ohio* 26 2349–2360. 10.1634/stemcells.2008-0080 18556510 PMC2649803

[B33] Delgado A. C. Maldonado-Soto A. R. Silva-Vargas V. Mizrak D. von Känel T. Tan K. R. (2021). Release of stem cells from quiescence reveals gliogenic domains in the adult mouse brain. *Science* 372 1205–1209. 10.1126/science.abg8467 34112692

[B34] Deneen B. Ho R. Lukaszewicz A. Hochstim C. J. Gronostajski R. M. Anderson D. J. (2006). The transcription factor NFIA controls the onset of gliogenesis in the developing spinal cord. *Neuron* 52 953–968. 10.1016/j.neuron.2006.11.019 17178400

[B35] El Waly B. Macchi M. Cayre M. Durbec P. (2014). Oligodendrogenesis in the normal and pathological central nervous system. *Front. Neurosci.* 8:145. 10.3389/fnins.2014.00145 24971048 PMC4054666

[B36] Etxeberria A. Mangin J.-M. Aguirre A. Gallo V. (2010). Adult-born SVZ progenitors receive transient glutamatergic synapses during remyelination of the corpus callosum. *Nat. Neurosci.* 13 287–289. 10.1038/nn.2500 20173746 PMC4681435

[B37] Faiz M. Sachewsky N. Gascón S. Bang K. W. A. Morshead C. M. Nagy A. (2015). Adult neural stem cells from the subventricular zone give rise to reactive astrocytes in the cortex after stroke. *Cell Stem Cell* 17 624–634. 10.1016/j.stem.2015.08.002 26456685

[B38] Gadea A. Aguirre A. Haydar T. F. Gallo V. (2009). Endothelin-1 regulates oligodendrocyte development. *J. Neurosci. Off. J. Soc. Neurosci.* 29 10047–10062. 10.1523/JNEUROSCI.0822-09.2009 19675238 PMC2754292

[B39] Gill D. Veltkamp R. (2016). Dynamics of T cell responses after stroke. *Curr. Opin. Pharmacol.* 26 26–32. 10.1016/j.coph.2015.09.009 26452204

[B40] Girard F. Eichenberger S. Celio M. R. (2014). Thrombospondin 4 deficiency in mouse impairs neuronal migration in the early postnatal and adult brain. *Mol. Cell. Neurosci.* 61 176–186. 10.1016/j.mcn.2014.06.010 24983516

[B41] Givogri M. I. de Planell M. Galbiati F. Superchi D. Gritti A. Vescovi A. (2006). Notch signaling in astrocytes and neuroblasts of the adult subventricular zone in health and after cortical injury. *Dev. Neurosci.* 28 81–91. 10.1159/000090755 16508306

[B42] Goings G. E. Sahni V. Szele F. G. (2004). Migration patterns of subventricular zone cells in adult mice change after cerebral cortex injury. *Brain Res.* 996 213–226. 10.1016/j.brainres.2003.10.034 14697499

[B43] Gomes W. A. Mehler M. F. Kessler J. A. (2003). Transgenic overexpression of BMP4 increases astroglial and decreases oligodendroglial lineage commitment. *Dev. Biol.* 255 164–177. 10.1016/s0012-1606(02)00037-4 12618141

[B44] Gonzalez-Perez O. Alvarez-Buylla A. (2011). Oligodendrogenesis in the subventricular zone and the role of epidermal growth factor. *Brain Res. Rev.* 67 147–156. 10.1016/j.brainresrev.2011.01.001 21236296 PMC3109119

[B45] Gonzalez-Perez O. Romero-Rodriguez R. Soriano-Navarro M. Garcia-Verdugo J. M. Alvarez-Buylla A. (2009). Epidermal growth factor induces the progeny of subventricular zone type b cells to migrate and differentiate into oligodendrocytes. *Stem Cells* 27 2032–2043. 10.1002/stem.119 19544429 PMC3346259

[B46] Grelat A. Benoit L. Wagner S. Moigneu C. Lledo P.-M. Alonso M. (2018). Adult-born neurons boost odor–reward association. *Proc. Natl. Acad. Sci. U.S.A.* 115 2514–2519. 10.1073/pnas.1716400115 29467284 PMC5877928

[B47] Gross R. E. Mehler M. F. Mabie P. C. Zang Z. Santschi L. Kessler J. A. (1996). Bone morphogenetic proteins promote astroglial lineage commitment by mammalian subventricular zone progenitor cells. *Neuron* 17 595–606. 10.1016/S0896-6273(00)80193-2 8893018

[B48] Guardia Clausi M. Paez P. M. Campagnoni A. T. Pasquini L. A. Pasquini J. M. (2012). Intranasal administration of aTf protects and repairs the neonatal white matter after a cerebral hypoxic–ischemic event. *Glia* 60, 1540–1554. 10.1002/glia.22374 22736466

[B49] Hammond T. R. McEllin B. Morton P. D. Raymond M. Dupree J. Gallo V. (2015). Endothelin-B receptor activation in astrocytes regulates the rate of oligodendrocyte regeneration during remyelination. *Cell Rep.* 13 2090–2097. 10.1016/j.celrep.2015.11.002 26628380

[B50] Huang A. Y.-S. Woo J. Sardar D. Lozzi B. Bosquez Huerta N. A. Lin C.-C. J. (2020). Region-specific transcriptional control of astrocyte function oversees local circuit activities. *Neuron* 106 992.e9–1008.e9. 10.1016/j.neuron.2020.03.025 32320644 PMC7879989

[B51] Huang S. Choi M. H. Huang H. Wang X. Chang Y. C. Kim J. Y. (2020). Demyelination regulates the circadian transcription factor BMAL1 to signal adult neural stem cells to initiate oligodendrogenesis. *Cell Rep.* 33:108394. 10.1016/j.celrep.2020.108394 33207207

[B52] Ihrie R. A. Álvarez-Buylla A. (2011). Lake front property: a unique germinal niche by the lateral ventricles of the adult brain. *Neuron* 70 674–686. 10.1016/j.neuron.2011.05.004 21609824 PMC3346178

[B53] Jablonska B. Scafidi J. Aguirre A. Vaccarino F. Nguyen V. Borok E. (2012). Oligodendrocyte regeneration after neonatal hypoxia requires FoxO1-mediated p27Kip1 expression. *J. Neurosci. Off. J. Soc. Neurosci.* 32 14775–14793. 10.1523/JNEUROSCI.2060-12.2012 23077062 PMC3517297

[B54] Jadasz J. J. Rivera F. J. Taubert A. Kandasamy M. Sandner B. Weidner N. (2012). p57kip2 regulates glial fate decision in adult neural stem cells. *Dev. Camb. Engl.* 139 3306–3315. 10.1242/dev.074518 22874918

[B55] John Lin C.-C. Yu K. Hatcher A. Huang T.-W. Lee H. K. Carlson J. (2017). Identification of diverse astrocyte populations and their malignant analogs. *Nat. Neurosci.* 20 396–405. 10.1038/nn.4493 28166219 PMC5824716

[B56] Jones T. A. (2017). Motor compensation and its effects on neural reorganization after stroke. *Nat. Rev. Neurosci.* 18 267–280. 10.1038/nrn.2017.26 28331232 PMC6289262

[B57] Kako E. Kaneko N. Aoyama M. Hida H. Takebayashi H. Ikenaka K. (2012). Subventricular zone-derived oligodendrogenesis in injured neonatal white matter in mice enhanced by a nonerythropoietic erythropoietin derivative. *Stem Cells* 30 2234–2247. 10.1002/stem.1202 22890889

[B58] Kang P. Lee H. K. Glasgow S. M. Finley M. Donti T. Gaber Z. B. (2012). Sox9 and NFIA coordinate a transcriptional regulatory cascade during the initiation of gliogenesis. *Neuron* 74 79–94. 10.1016/j.neuron.2012.01.024 22500632 PMC3543821

[B59] Kang W. Nguyen K. C. Q. Hébert J. M. (2019). Transient redirection of SVZ stem cells to oligodendrogenesis by FGFR3 activation promotes remyelination. *Stem Cell Rep.* 12 1223–1231. 10.1016/j.stemcr.2019.05.006 31189094 PMC6565886

[B60] Kazanis I. Evans K. A. Andreopoulou E. Dimitriou C. Koutsakis C. Karadottir R. T. (2017). Subependymal zone-derived oligodendroblasts respond to focal demyelination but fail to generate myelin in young and aged mice. *Stem Cell Rep.* 8 685–700. 10.1016/j.stemcr.2017.01.007 28196689 PMC5355571

[B61] Kernie S. G. Erwin T. M. Parada L. F. (2001). Brain remodeling due to neuronal and astrocytic proliferation after controlled cortical injury in mice. *J. Neurosci. Res.* 66 317–326. 10.1002/jnr.10013 11746349

[B62] Klingener M. Chavali M. Singh J. McMillan N. Coomes A. Dempsey P. J. (2014). N-cadherin promotes recruitment and migration of neural progenitor cells from the SVZ neural stem cell niche into demyelinated lesions. *J. Neurosci. Off. J. Soc. Neurosci.* 34 9590–9606. 10.1523/JNEUROSCI.3699-13.2014 25031401 PMC4099541

[B63] Lachapelle F. Avellana-Adalid V. Nait-Oumesmar B. Baron-Van Evercooren A. (2002). Fibroblast growth factor-2 (FGF-2) and platelet-derived growth factor AB (PDGF AB) promote adult SVZ-derived oligodendrogenesis in vivo. *Mol. Cell. Neurosci.* 20, 390–403. 10.1006/mcne.2002.1124 12139917

[B64] Laug D. Huang T.-W. Huerta N. A. B. Huang A. Y.-S. Sardar D. Ortiz-Guzman J. (2019). Nuclear factor I-A regulates diverse reactive astrocyte responses after CNS injury. *J. Clin. Invest.* 129 4408–4418. 10.1172/JCI127492 31498149 PMC6763246

[B65] Li L. Harms K. M. Ventura P. B. Lagace D. C. Eisch A. J. Cunningham L. A. (2010). Focal cerebral ischemia induces a multilineage cytogenic response from adult subventricular zone that is predominantly gliogenic. *Glia* 58 1610–1619. 10.1002/glia.21033 20578055 PMC2919586

[B66] Li W. Cao F. Takase H. Arai K. Lo E. H. Lok J. (2021). Blood–brain barrier mechanisms in stroke and trauma. *Handb. Exp. Pharmacol.* [Epub ahead of print]. 10.1007/164_2020_426 33580391

[B67] Li W. L. Chu M. W. Wu A. Suzuki Y. Imayoshi I. Komiyama T. (2018). Adult-born neurons facilitate olfactory bulb pattern separation during task engagement. *eLife* 7:e33006. 10.7554/eLife.33006 29533179 PMC5912906

[B68] Liesz A. Dalpke A. Mracsko E. Roth S. Zhou W. Yang H. (2015). DAMP signaling is a key pathway inducing immune modulation after brain injury. *J. Neurosci.* 35 583–598. 10.1523/JNEUROSCI.2439-14.2015 25589753 PMC4293412

[B69] Lim D. A. Tramontin A. D. Trevejo J. M. Herrera D. G. García-Verdugo J. M. Alvarez-Buylla A. (2000). Noggin antagonizes BMP signaling to create a niche for adult neurogenesis. *Neuron* 28 713–726. 10.1016/s0896-6273(00)00148-3 11163261

[B70] Lin S. Fan L.-W. Pang Y. Rhodes P. G. Mitchell H. J. Cai Z. (2005). IGF-1 protects oligodendrocyte progenitor cells and improves neurological functions following cerebral hypoxia-ischemia in the neonatal rat. *Brain Res.* 1063 15–26. 10.1016/j.brainres.2005.09.042 16259966

[B71] Liu B. F. Gao E. J. Zeng X. Z. Ji M. Cai Q. Lu Q. (2006). Proliferation of neural precursors in the subventricular zone after chemical lesions of the nigrostriatal pathway in rat brain. *Brain Res.* 1106 30–39. 10.1016/j.brainres.2006.05.111 16843444

[B72] Lois C. Alvarez-Buylla A. (1994). Long-distance neuronal migration in the adult mammalian brain. *Science* 264 1145–1148. 10.1126/science.8178174 8178174

[B73] Lois C. García-Verdugo J. M. Alvarez-Buylla A. (1996). Chain migration of neuronal precursors. *Science* 271 978–981. 10.1126/science.271.5251.978 8584933

[B74] Luo F. Zhang Z. Barnett A. Bellinger T. J. Turcato F. Schmidt K. (2020). Cuprizone-induced demyelination under physiological and post-stroke condition leads to decreased neurogenesis response in adult mouse brain. *Exp. Neurol.* 326:113168. 10.1016/j.expneurol.2019.113168 31904386 PMC9694109

[B75] Maki T. Liang A. Miyamoto N. Lo E. Arai K. (2013). Mechanisms of oligodendrocyte regeneration from ventricular-subventricular zone-derived progenitor cells in white matter diseases. *Front. Cell. Neurosci.* 7:275. 10.3389/fncel.2013.00275 24421755 PMC3872787

[B76] Martini S. Bernoth K. Main H. Ortega G. D. C. Lendahl U. Just U. (2013). A critical role for Sox9 in notch-induced astrogliogenesis and stem cell maintenance. *Stem Cells* 31 741–751. 10.1002/stem.1320 23307615

[B77] Martín-López E. García-Marques J. Núñez-Llaves R. López-Mascaraque L. (2013). Clonal astrocytic response to cortical injury. *PLoS One* 8:e74039. 10.1371/journal.pone.0074039 24040158 PMC3769363

[B78] Menn B. Garcia-Verdugo J. M. Yaschine C. Gonzalez-Perez O. Rowitch D. Alvarez-Buylla A. (2006). Origin of oligodendrocytes in the subventricular zone of the adult brain. *J. Neurosci.* 26 7907–7918. 10.1523/JNEUROSCI.1299-06.2006 16870736 PMC6674207

[B79] Merkle F. T. Mirzadeh Z. Alvarez-Buylla A. (2007). Mosaic organization of neural stem cells in the adult brain. *Science* 317 381–384. 10.1126/science.1144914 17615304

[B80] Mizrak D. Levitin H. M. Delgado A. C. Crotet V. Yuan J. Chaker Z. (2019). Single-cell analysis of regional differences in adult V-SVZ neural stem cell lineages. *Cell Rep.* 26 394.e5–406.e5. 10.1016/j.celrep.2018.12.044 30625322 PMC6368857

[B81] Molofsky A. V. Krenick R. Ullian E. Tsai H. Deneen B. Richardson W. D. (2012). Astrocytes and disease: a neurodevelopmental perspective. *Genes Dev.* 26 891–907. 10.1101/gad.188326.112 22549954 PMC3347787

[B82] Morel L. Chiang M. S. R. Higashimori H. Shoneye T. Iyer L. K. Yelick J. (2017). Molecular and functional properties of regional astrocytes in the adult brain. *J. Neurosci.* 37 8706–8717. 10.1523/JNEUROSCI.3956-16.2017 28821665 PMC5588463

[B83] Morell M. Tsan Y. O’Shea K. S. (2015). Inducible expression of noggin selectively expands neural progenitors in the adult SVZ. *Stem Cell Res.* 14 79–94. 10.1016/j.scr.2014.11.001 25535864

[B84] Mundim M. V. Zamproni L. N. Pinto A. A. S. Galindo L. T. Xavier A. M. Glezer I. (2019). A new function for Prokineticin 2: recruitment of SVZ-derived neuroblasts to the injured cortex in a mouse model of traumatic brain injury. *Mol. Cell. Neurosci.* 94 1–10. 10.1016/j.mcn.2018.10.004 30391355

[B85] Murcia-Belmonte V. Esteban P. F. Martínez-Hernández J. Gruart A. Luján R. Delgado-García J. M. (2016). Anosmin-1 over-expression regulates oligodendrocyte precursor cell proliferation, migration and myelin sheath thickness. *Brain Struct. Funct.* 221 1365–1385. 10.1007/s00429-014-0977-4 25662897

[B86] Nait-Oumesmar B. Decker L. Lachapelle F. Avellana-Adalid V. Bachelin C. Baron-Van Evercooren A. (1999). Progenitor cells of the adult mouse subventricular zone proliferate, migrate and differentiate into oligodendrocytes after demyelination. *Eur. J. Neurosci.* 11 4357–4366. 10.1046/j.1460-9568.1999.00873.x 10594662

[B87] Nait-Oumesmar B. Picard-Riera N. Kerninon C. Decker L. Seilhean D. Höglinger G. U. (2007). Activation of the subventricular zone in multiple sclerosis: evidence for early glial progenitors. *Proc. Natl. Acad. Sci. U.S.A.* 104 4694–4699. 10.1073/pnas.0606835104 17360586 PMC3025281

[B88] Namihira M. Kohyama J. Semi K. Sanosaka T. Deneen B. Taga T. (2009). Committed neuronal precursors confer astrocytic potential on residual neural precursor cells. *Dev. Cell* 16 245–255. 10.1016/j.devcel.2008.12.014 19217426

[B89] Okada S. Nakamura M. Katoh H. Miyao T. Shimazaki T. Ishii K. (2006). Conditional ablation of Stat3 or Socs3 discloses a dual role for reactive astrocytes after spinal cord injury. *Nat. Med.* 12 829–834. 10.1038/nm1425 16783372

[B90] Ortega F. Gascón S. Masserdotti G. Deshpande A. Simon C. Fischer J. (2013). Oligodendrogliogenic and neurogenic adult subependymal zone neural stem cells constitute distinct lineages and exhibit differential responsiveness to Wnt signalling. *Nat. Cell Biol.* 15 602–613. 10.1038/ncb2736 23644466

[B91] Pekny M. Pekna M. Messing A. Steinhäuser C. Lee J.-M. Parpura V. (2016). Astrocytes: a central element in neurological diseases. *Acta Neuropathol.* 131 323–345. 10.1007/s00401-015-1513-1 26671410

[B92] Pekny M. Wilhelmsson U. Tatlisumak T. Pekna M. (2019). Astrocyte activation and reactive gliosis-A new target in stroke? *Neurosci. Lett.* 689 45–55. 10.1016/j.neulet.2018.07.021 30025833

[B93] Picard-Riera N. Decker L. Delarasse C. Goude K. Nait-Oumesmar B. Liblau R. (2002). Experimental autoimmune encephalomyelitis mobilizes neural progenitors from the subventricular zone to undergo oligodendrogenesis in adult mice. *Proc. Natl. Acad. Sci. U.S.A.* 99 13211–13216. 10.1073/pnas.192314199 12235363 PMC130612

[B94] Pinto L. I. G. Rodríguez D. Adamo A. M. Mathieu P. A. (2018). TGF-β pro-oligodendrogenic effects on adult SVZ progenitor cultures and its interaction with the Notch signaling pathway. *Glia* 66 396–412. 10.1002/glia.23253 29076551

[B95] Pous L. Deshpande S. S. Nath S. Mezey S. Malik S. C. Schildge S. (2020). Fibrinogen induces neural stem cell differentiation into astrocytes in the subventricular zone via BMP signaling. *Nat. Commun.* 11:630. 10.1038/s41467-020-14466-y 32005867 PMC6994610

[B96] Radomski K. L. Zhou Q. Yi K. J. Doughty M. L. (2013). Cortical contusion injury disrupts olfactory bulb neurogenesis in adult mice. *BMC Neurosci.* 14:142. 10.1186/1471-2202-14-142 24224996 PMC3830448

[B97] Remaud S. Ortiz F. C. Perret-Jeanneret M. Aigrot M.-S. Gothié J.-D. Fekete C. (2017). Transient hypothyroidism favors oligodendrocyte generation providing functional remyelination in the adult mouse brain. *eLife* 6:e29996. 10.7554/eLife.29996 28875931 PMC5779229

[B98] Saha B. Peron S. Murray K. Jaber M. Gaillard A. (2013). Cortical lesion stimulates adult subventricular zone neural progenitor cell proliferation and migration to the site of injury. *Stem Cell Res.* 11 965–977. 10.1016/j.scr.2013.06.006 23900166

[B99] Scafidi J. Hammond T. R. Scafidi S. Ritter J. Jablonska B. Roncal M. (2014). Intranasal epidermal growth factor treatment rescues neonatal brain injury. *Nature* 506 230–234. 10.1038/nature12880 24390343 PMC4106485

[B100] Serwanski D. R. Rasmussen A. L. Brunquell C. B. Perkins S. S. Nishiyama A. (2018). Sequential contribution of parenchymal and neural stem cell-derived oligodendrocyte precursor cells toward remyelination. *Neuroglia Basel Switz.* 1 91–105. 10.3390/neuroglia1010008 30662979 PMC6335037

[B101] Sloan S. A. Barres B. A. (2014). Mechanisms of astrocyte development and their contributions to neurodevelopmental disorders. *Curr. Opin. Neurobiol.* 27 75–81. 10.1016/j.conb.2014.03.005 24694749 PMC4433289

[B102] Sofroniew M. V. (2020). Astrocyte reactivity: subtypes, states, and functions in CNS innate immunity. *Trends Immunol.* 41 758–770. 10.1016/j.it.2020.07.004 32819810 PMC7484257

[B103] Sohn J. Orosco L. Guo F. Chung S.-H. Bannerman P. Mills (2015). The subventricular zone continues to generate corpus callosum and rostral migratory stream astroglia in normal adult mice. *J. Neurosci. Off. J. Soc. Neurosci.* 35 3756–3763. 10.1523/JNEUROSCI.3454-14.2015 25740506 PMC6605576

[B104] Song H. W. Kumar B. K. Kim S. H. Jeon Y. H. Lee Y. A. Lee W. T. (2011). Agmatine enhances neurogenesis by increasing ERK1/2 expression, and suppresses astrogenesis by decreasing BMP 2,4 and SMAD 1,5,8 expression in subventricular zone neural stem cells. *Life Sci.* 89 439–449. 10.1016/j.lfs.2011.07.003 21843531

[B105] Soundarapandian M. M. Selvaraj V. Lo U.-G. Golub M. S. Feldman D. H. Pleasure D. E. (2011). Zfp488 promotes oligodendrocyte differentiation of neural progenitor cells in adult mice after demyelination. *Sci. Rep.* 1:2. 10.1038/srep00002 22355521 PMC3210692

[B106] Stolt C. C. Lommes P. Sock E. Chaboissier M.-C. Schedl A. Wegner M. (2003). The Sox9 transcription factor determines glial fate choice in the developing spinal cord. *Genes Dev.* 17 1677–1689. 10.1101/gad.259003 12842915 PMC196138

[B107] Sullivan G. M. Mierzwa A. J. Kijpaisalratana N. Tang H. Wang Y. Song S.-K. (2013). Oligodendrocyte lineage and subventricular zone response to traumatic axonal injury in the corpus callosum. *J. Neuropathol. Exp. Neurol.* 72 1106–1125. 10.1097/NEN.0000000000000009 24226267 PMC4130339

[B108] Sun W. Cornwell A. Li J. Peng S. Osorio M. J. Aalling N. (2017). SOX9 is an astrocyte-specific nuclear marker in the adult brain outside the neurogenic regions. *J. Neurosci.* 37 4493–4507. 10.1523/JNEUROSCI.3199-16.2017 28336567 PMC5413187

[B109] Tong C. K. Fuentealba L. C. Shah J. K. Lindquist R. A. Ihrie R. A. Guinto C. D. (2015). A dorsal SHH-dependent domain in the V-SVZ produces large numbers of oligodendroglial lineage cells in the postnatal brain. *Stem Cell Rep.* 5 461–470. 10.1016/j.stemcr.2015.08.013 26411905 PMC4624995

[B110] Ung K. Huang T.-W. Lozzi B. Woo J. Hanson E. Pekarek B. (2021). Olfactory bulb astrocytes mediate sensory circuit processing through Sox9 in the mouse brain. *Nat. Commun.* 12:5230. 10.1038/s41467-021-25444-3 34471129 PMC8410770

[B111] Vernerey J. Macchi M. Magalon K. Cayre M. Durbec P. (2013). Ciliary neurotrophic factor controls progenitor migration during remyelination in the adult rodent brain. *J. Neurosci.* 33 3240–3250. 10.1523/JNEUROSCI.2579-12.2013 23407977 PMC6619230

[B112] Wang Z. Zheng Y. Zheng M. Zhong J. Ma F. Zhou B. (2019). Neurogenic niche conversion strategy induces migration and functional neuronal differentiation of neural precursor cells following brain injury. *Stem Cells Dev.* 29 235–248. 10.1089/scd.2019.0147 31797735

[B113] Xing Y. L. Röth P. T. Stratton J. A. S. Chuang B. H. A. Danne J. Ellis S. L. (2014). Adult neural precursor cells from the subventricular zone contribute significantly to oligodendrocyte regeneration and remyelination. *J. Neurosci. Off. J. Soc. Neurosci.* 34 14128–14146. 10.1523/JNEUROSCI.3491-13.2014 25319708 PMC6705285

[B114] Xu L. Sapolsky R. M. Giffard R. G. (2001). Differential sensitivity of murine astrocytes and neurons from different brain regions to injury. *Exp. Neurol.* 169 416–424. 10.1006/exnr.2001.7678 11358455

[B115] Yang Z. Levison S. W. (2006). Hypoxia/ischemia expands the regenerative capacity of progenitors in the perinatal subventricular zone. *Neuroscience* 139 555–564. 10.1016/j.neuroscience.2005.12.059 16500031

[B116] Young K. M. Fogarty M. Kessaris N. Richardson W. D. (2007). Subventricular zone stem cells are heterogeneous with respect to their embryonic origins and neurogenic fates in the adult olfactory bulb. *J. Neurosci. Off. J. Soc. Neurosci.* 27 8286–8296. 10.1523/JNEUROSCI.0476-07.2007 17670975 PMC6331046

[B117] Zaidi A. U. Bessert D. A. Ong J. E. Xu H. Barks J. D. E. Silverstein F. S. (2004). New oligodendrocytes are generated after neonatal hypoxic-ischemic brain injury in rodents. *Glia* 46 380–390. 10.1002/glia.20013 15095368

[B118] Zhong J. Zhao L. Du Y. Wei G. Yao W.-G. Lee W.-H. (2009). Delayed IGF-1 treatment reduced long-term hypoxia-ischemia-induced brain damage and improved behavior recovery of immature rats. *Neurol. Res.* 31 483–489. 10.1179/174313208X338133 19500451

[B119] Zilkha-Falb R. Gurevich M. Hanael E. Achiron A. (2017). Prickle1 as positive regulator of oligodendrocyte differentiation. *Neuroscience* 364 107–121. 10.1016/j.neuroscience.2017.09.018 28935237

